# Intellectual disability and nutrition‐related health

**DOI:** 10.15252/emmm.202012899

**Published:** 2020-08-19

**Authors:** Svein O Kolset

**Affiliations:** ^1^ Department of Nutrition University of Oslo Oslo Norway

**Keywords:** Genetics, Gene Therapy & Genetic Disease, Metabolism

## Abstract

Intellectual disability (ID) is a condition that affects approximately 1% of the population (Maulik *et al*, 2011). The numbers may differ across nations, owing to different systems and diagnosis entries or lack of such, but usually range between 0.6 and 3% (Stromme & Valvatne, 1998). Persons with ID are a heterogeneous group with different diagnoses and different levels of intellectual ability. These range from profound (IQ < 20) and serious ID (IQ 20–34) to moderate (IQ 35–49) and light ID (IQ 50–69); this roughly translates into the intellectual capacity of children between 3–12 years of age. More than 75% of persons with ID have the mild form and their intellectual capacity and potential may be underestimated in some cases if IQ is the only diagnostic criteria. However, the range in itself is an important factor to take into account when addressing nutrition and health issues. It is further important to recognize that ID is also a feature of several rare disorders, and many disorders not yet identified, adding to the complexity of this group.

## Graphical abstract

In this Commentary, S. Kolset discusses the complex interplay between intellectual disability (ID) and nutrition, and presents the scientific and policy challenges that need to be addressed to improve health for persons with ID.

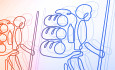

Generally, there are several common characteristics among the diverse phenotypic presentations of ID that impact health and nutrition:

• Impulsive behavior and difficulties in controlling emotions.
• Comorbid conditions.
• Psychiatric issues and effects of medication on weight and eating behavior.
• Difficulties in focusing on multiple issues.
• Lack of physical activity.
• Challenges linked to speed of events and flow of information.
• Need for life‐long learning.

## Causes

Genetic causes of ID are increasingly being studied in several countries (Wang, Wang *et al*, 2020), but external factors such as infection, alcohol exposure during pregnancy, inborn errors of metabolism, and exposure to pollutants also play a role. Down syndrome is the best known and one of the most studied forms of genetically caused ID, and a considerable percentage of persons with autism spectrum disorders (ASD) have ID. ID is also a prominent feature in several other disorders, such as Fragile X syndrome, Rett syndrome, Prader–Willi syndrome, and William syndrome.

Advances in sequencing technology and increased interest in ID have improved our knowledge on the genetics, despite the methodological challenges that come with the genetic heterogeneity between the different groups. Changes in more than 700 genes have been linked to ID and ID‐associated disorders, and *de novo* mutations and copy number variants are important risk factors (Bass & Skuse, 2018). Many of these changes link ID to other neurodevelopmental disorders such as attention deficit hyperactivity disorder or ASD. Several of the identified genetic changes modify the activities of proteins involved in signal transduction, transcriptional regulation and neurodevelopment. In the future, animal models will be of great use to provide an in‐depth understanding of the development of ID, such as the role of transcription factors recently documented for Pitt–Hopkins syndrome (Schoof *et al*, 2020).

Studies of underlying molecular mechanisms may have an impact on the lives of those affected. One example is cerebral creatine deficiency, an inborn error of metabolism, which can lead to several conditions with ID. Some forms of this disease are treatable with oral supplementation to replenish creatine levels in the brain. Another example is phenylketonuria, a disease that, if untreated, will lead to ID. It is also an important example from a nutritional point of view, as the treatment is to avoid phenylalanine in the diet. Screening of newborns allows early detection and treatment, but is unfortunately not standard procedure in all countries. Several other inborn errors of metabolism linked to ID have been identified and an app with diagnostic tools and suggested therapies has been published (van Karnebeek *et al*, 2014). Treating these preventable or treatable forms of ID require the support of experts in nutrition and medicine.

## Nutrition

In these more rare diseases, ID is usually an outcome of metabolic errors, and nutrition and a special diet are needed to restore and maintain a normal metabolism. In contrast, nutrition research has documented that many persons with ID, especially individuals with mild and moderate ID, become overweight with age and suffer from several different comorbidities (Sandberg *et al*, 2017). Here, the aim is to prevent the onset of obesity or malnutrition in persons with ID through promoting a healthy diet—which is also recommended for the general population. This poses various challenges for nutritionists and care givers.

On the contrary, there is a high level of under‐ or malnutrition in persons with profound or serious ID (Robertson *et al*, 2018). This has also been reported in persons with ASD with rigorous or “picky eater” patterns, and children with cerebral palsy or eating and swallowing problems.

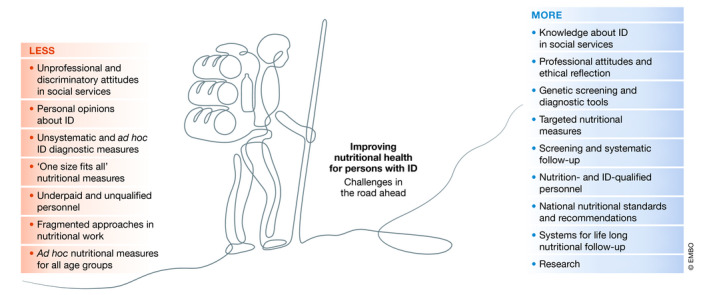

Nutritional measures and diet must be adjusted to the different syndromes and their inherent implications, which requires knowledge of ID in general, and of specific diagnosis groups in particular. From our experience, such knowledge is generally limited, both in health services and in the municipalities that provide daily care and other services (Hope *et al*, 2018). This is a serious challenge that should be addressed by scientists across specialties and nations.

Addressing the nutritional challenges of individuals with mild ID also requires collaboration between physicians, clinical nutritionists, psychologists, special‐education teachers, and physiotherapists. Challenging behavior, lower cognitive functions, and capacity in daily‐life activities translate into nutritional problems such as shopping, cooking and eating patterns, including snacking, that require systematic professional support. This support is to a large extent lacking, which calls for adapted screening tools, manuals, and systematic follow‐up. When working with persons with ID, it is important to recognize their need for life‐long assistance, a factor that is often underestimated.

From our own research, we also see the need for more intervention studies to investigate measures that are efficient for decreasing the prevalence of overweight in different subgroups of persons with ID. The development of adapted mobile phone programs (Polfuss *et al*, 2018) and apps will be of great value in interventions, educational studies and for persons with ID to help them managing their daily chores and their diet, particularly those with moderate and light ID. These are challenges that demand expertise in technology, pedagogics, health care, and many other areas.

The wide range of phenotypes of ID also demonstrates the importance of using both qualitative and quantitative methods in research projects. There is an urgent need to increase the methodological repertoire in many types of ID studies and to encourage collaboration between different groups of professionals. The increasing attention to the link between nutrition and ID is reflected in several ways. In 2015, the Academy of Nutrition and Dietetics issued a position statement on the nutrition services for persons with ID (Ptomey & Wittenbrook, 2015). Dietary guidelines for treating phenylketonuria have been optimized and standardized across Europe. Revised diagnostic criteria for Rett syndrome have been published as well as recommendations for diagnosis and management of Prader–Willi syndrome following an international and multidisciplinary expert meeting. Improving nutritional health in persons with ID is in many ways a political question, but it is also the responsibility of the scientific communities.

## Policy challenges

Addressing the multifaceted challenges of nutrition and health in persons with ID requires more research and increased priority from funding agencies, along with increased visibility and knowledge of the various forms of ID in general. From both a molecular and medical perspective, several diseases involving ID pose challenges for research, such as regulation of feeding and satiety, which is an important issue in Prader–Willi syndrome. There are some positive changes in the scientific field in relation to ID. The increased attention is evident by looking at the number of publications within different research areas. Several EU research projects under Horizon 2020 on genetics and diagnosis on Rare disorders include ID (https://ec.europa.eu/info/research-and-innovation/research-area/health-research-and-innovation/rare-diseases_en). The heterogeneity of persons with ID makes it imperative for scientists to collaborate closely with patient organizations for recruitment, generation of well‐defined and relevant hypotheses, research agendas, and dissemination of results. Finally, the striking difference between clinical progress and societal stalemate has recently been addressed for persons with rare diseases; many of the points raised are equally important and relevant for persons with ID (Editorial, 2020).

In summary, measures to improve health for person with ID should include:

• More data on the number of persons with ID to address the extent and heterogeneity of their health challenges.
• Improved genetic screening to detect and define subgroups and hitherto unidentified groups with ID, also addressing age and gender issues and inborn errors of metabolism.
• Improved and refined methods to study people with ID, ranging from qualitative studies, epidemiology, nutritional screening to intervention studies, and molecular and mechanistic studies.
• Inclusion of persons with ID in national and cross‐national studies on cardiovascular diseases, cancer, and other diseases.
• More research to define disease mechanisms and neurodevelopmental disorders in different conditions with ID.
• Larger intervention studies to address overweight and comorbidity issues.
• Increased collaboration between scientists, granting organizations, and patient organizations.
• Collaborative research on ID involving nutrition, psychiatry, medicine, molecular biology, social work, physical activity, and pedagogics.

## Conflict of interest

The author declares that he has no conflict of interest.

## For more information

(i) Intellectual disability—NIH: https://www.nichd.nih.gov/health/topics/idds/conditioninfo/default
(ii) EU research on rare diseases: https://ec.europa.eu/info/research-and-innovation/research-area/health-research-and-innovation/rare-diseases_en
(iii) Rare diseases clinical research network: https://www.rarediseasesnetwork.org/
(iv) The portal for rare diseases and orphan drugs: https://www.orpha.net/consor/cgi-bin/index.php
(v) Society for inherited metabolic disorders: https://www.simd.org/Links/
